# Supplementary material for: Regular Periodic Surface Structures on Indium Tin Oxide Film Efficiently Fabricated by Femtosecond Laser Direct Writing with a Cylindrical Lens
Source: Materials (Basel). 2022 Jul 22;15(15):5092. doi: 10.3390/ma15155092 (PMC9329999; doi:10.3390/ma15155092)
Supplement: Supplementary file 1 [file materials-15-05092-s001.zip › materials-1776210-supplementary.pdf]

materials

MDPI

Supplement

Regular Periodic Surface Structures on Indium Tin Oxide Film Efficiently Fabricated by Femtosecond Laser Direct Writing with a Cylindrical Lens

Long Chen [1,ǂ], Jian Yang [1,ǂ], Qilin Jiang [1], Kaiqiang Cao [1], Jukun Liu [2,*], Tianqing Jia [1,*], Zhenrong Sun [1] and Hongxing Xu [1]

[1] State Key Laboratory of Precision Spectroscopy, School of Physics and Electronic Science, East China Normal University, Shanghai 200062, China; 1979612555@qq.com (L.C.); jyang@lps.ecnu.edu.cn (J.Y.); 389543058@qq.com (Q.L.); caokq1010@163.com (K.Q.); zrsun@phy.ecnu.edu.cn (Z.R.); hxxu@whu.edu.cn (H.X.)
[2] College of Science, Shanghai Institute of Technology, Shanghai 201418, P.R. China
* Correspondence: liujukun@126.com; tqjia@phy.ecnu.edu.cn
ǂ The two authors made equal contributions.

Supplement

The dielectric permittivity of ITO in the ground state is 3.6159 + 0.0085i, which is taken from reference [1]. The dielectric permittivity of ITO in the excited state is calculated to be -4.45 + 0.35i according to the Drude model, in which the carrier density is calculated from the Boltzmann's transport equations [2, 3].

The dielectric function $\varepsilon_r(\omega)$ is related to the optical properties of the material and the frequency of the incident laser. Here, the Drude model is used to describe the optical properties of ITO in the excited state, which is expressed by the following equations:

$$\varepsilon_r(\omega) = \varepsilon - \frac{\omega_p^2}{\omega(\omega + i v_c)}, \tag{S1}$$

$$\omega_p = \sqrt{e^2 N_c / (\varepsilon_0 m_{opt} m_e)}, \tag{S2}$$

Here, $\varepsilon_r(\omega)$ is the dielectric function of ITO in the excited state as a function of the laser angular frequency ω, v_c is the plasma collision frequency, m_{opt} is the optical mass of the carrier, ε is the optical dielectric constant of ITO in the ground state, ε_0 is the permittivity for free space, ω_p is the bulk plasma frequency, and N_c is the carrier density which is an important parameter determining the optical properties of materials. We can simply obtain the carrier density under different laser fluences by solving the one-dimensional Boltzmann's transport equation [2]:

$$\frac{\partial N_c}{\partial t} = D_0 \frac{\partial}{\partial x}\left(\frac{\partial N_c}{\partial x}\right) - \gamma N_c^3 + \delta N_c + \frac{\beta F^2}{2\hbar\omega}, \tag{S3}$$

where D_0 is the bipolar diffusion coefficient which is related to electron hole mobility and electron temperature, γ is the Auger recombination coefficient, δ is the collision ionization coefficient, β is the two-photon absorption coefficient, and $\hbar$ is Planck's constant divided by 2π.

References

1. Moerland, R.J.; Hoogenboom. J.P. Subnanometer-accuracy optical distance ruler based on fluorescence quenching by transparent conductors. *Optica* **2016**, *3*, 12-116.
2. Liu J.K.; Jia X.; Wu W.S.; Cheng K.; Feng D.H.; Zhang S.A.; Sun Z.R.; Jia T.Q. Ultrafast imaging on the formation of periodic ripples on a Si surface with a prefabricated nanogroove induced by a single femtosecond laser pulse. *Opt. Express* **2018**, *26*, 6302-6315.
3. Ederth, J.; Johnsson, P.; Niklasson, G.A.; Hoel, A.; Hultåker, A.; Heszler, P.; Granqvist, C.G.; Van Doorn, A.R.; Jongerius, M.J.; Burgard, D. Electrical and optical properties of thin films consisting of tin-doped indium oxide nanoparticles. *Phys. Rev. B* **2003**, *68*.
